# Effects of Solid Fermentation on *Polygonatum cyrtonema* Polysaccharides: Isolation, Characterization and Bioactivities

**DOI:** 10.3390/molecules28145498

**Published:** 2023-07-19

**Authors:** Yi Cheng, Xueyuan Huang, Lixia Li, Lu Liu, Chunsheng Zhang, Xiang Fan, Yu Xie, Yuanfeng Zou, Zhe Geng, Chao Huang

**Affiliations:** 1 Department of Physical Education, Chengdu University of Information Technology, Chengdu 611130, China; chengyi@cuit.edu.cn (Y.C.); liulu@cuit.edu.cn (L.L.); zcsheng2004@cuit.edu.cn (C.Z.); fangd@cuit.edu.cn (X.F.); xieyu@cuit.edu.cn (Y.X.); 2 Key Laboratory of Animal Disease and Human Health of Sichuan Province, College of Veterinary Medicine, Sichuan Agricultural University, Chengdu 611130, China; huangxueyuan1999@163.com (X.H.); lilixia905@163.com (L.L.); yuanfengzou@sicau.edu.cn (Y.Z.); 3 Natural Medicine Research Center, College of Veterinary Medicine, Sichuan Agricultural University, Chengdu 611130, China

**Keywords:** Polygonati Rhizoma, fermentation, polysaccharide, antioxidant, immunomodulation, gut microbiota

## Abstract

Polygonati Rhizoma is a widely used traditional Chinese medicine (TCM) with complex pre-processing steps. Fermentation is a common method for processing TCM to reduce herb toxicity and enhance their properties and/or produce new effects. Here, in this study, using *Bacillus subtilis* and *Saccharomyces cerevisiae*, we aimed to evaluate the potential application of solid fermentation in isolating different functional polysaccharides from *Polygonatum cyrtonema* Hua. With hot water extraction, ethanol precipitation, DEAE anion exchange chromatography and gel filtration, multiple neutral and acidic polysaccharides were obtained, showing different yields, content, compositions and functional groups after fermentation. Combining in vitro experiments and in vivo aging and immunosuppressed mouse models, we further compared the antioxidant and immunomodulating bioactivities of these polysaccharides and found a prominent role of a natural polysaccharide (BNP) from fermented *P. cyrtonema* via *Bacillus subtilis* in regulating intestinal antioxidant defense and immune function, which may be a consequence of the ability of BNP to modulate the homeostasis of gut microbiota. Thus, this work provides evidence for the further development and utilization of *P. cyrtonema* with fermentation, and reveals the potential values of BNP in the treatment of intestinal disorders.

## 1. Introduction

Polygonati Rhizoma is a traditional Chinese medicine (TCM) obtained from the rhizome of *Liliaceae Polygonatum* plants, including *Polygonatum sibiricum* Red, *Polygonatum cyrtonema* Hua, and *Polygonatum kingianum* Coll. et Hemsl, according to Chinese Pharmacopoeia, all of which are widely distributed and have been used as food and medicine for thousands of years in East Asia [1,2,3]. Lots of pharmacological effects, such as immune improvement, anti-aging, anti-diabetes and anti-fatigue, anti-cancer and anti-heart disease activities, have been reported of Polygonati Rhizoma [3,4], making studies on its bioactive compounds attract growing interest. While the compounds enriched in Polygonati Rhizoma are complex and the steroidal saponins, triterpenes, flavones, phytosterol and volatile oils from Polygonati Rhizoma display effective bioactive effects, polysaccharides are thought to be one of the most important bioactive compounds deriving from it. For example, multiple studies have demonstrated the immunomodulation and antioxidative effects of Polygonati Rhizoma polysaccharide [5,6,7,8,9], as well as the effects of anti-fatigue, the prevention of osteoporosis and Alzheimer’s disease, anti-diabetic and anti-cancer properties and so on [10]. Therefore, method improvements for better polysaccharide isolation from Polygonati Rhizoma could bring about benefits for its further development and utilization.

Fermentation is one of the most important processing methods in traditional Chinese medicine, whereby herbs are fermented under different conditions based on microorganisms to enhance their original properties and/or produce new effects [11]. Fermentation technology has been applied to processing lots of traditional Chinese medicine, like goji, *Pinellia Ternata* and *Rhei Radix et Rhizoma*, for thousands of years, and it can reduce herb toxicity and improve its efficacy [12]. Nowadays, according to the state of the fermentation medium, two different fermentation technologies—liquid and solid fermentation—are used in traditional Chinese medicine processing, and solid fermentation is the most traditional one [11,13]. The traditional processing method of Polygonati Rhizoma is “nine-steam-nine-bask” [14], which is time-consuming and laborious. Wang et al. reported that the fermentation of Polygonati Rhizoma produced more flavor and functional substances [15], and Hu et al. found that fermentation changed the content of bioactive constituents in Polygonati Rhizoma [16], suggesting that fermentation may be an available method for Polygonati Rhizoma processing. However, it is currently unclear how fermentation will affect the yield, content, structure and bioactivities of *P. cyrtonema* polysaccharide. Therefore, in this study, we aimed to study the effects of fermentation on the isolation and bioactivities of *P. cyrtonema* polysaccharide, with a solid fermentation method using *Polygonatum cyrtonema* Hua that is most widely planted in China and two commonly used bacterial and yeast strains, *Bacillus subtilis* and *Saccharomyces cerevisiae*, to provide evidence for the further development and utilization of Polygonati Rhizoma.

## 2. Results

### 2.1. Extraction, Purification and Preliminary Characterization of P. cyrtonema Polysaccharide

#### 2.1.1. Extraction of Crude Polysaccharides

The raw polysaccharides from the different herbs were obtained from water extracts after ethanol precipitation. Table 1 shows the extraction rate and components of raw polysaccharides, and it can be noted that the extraction rate of raw polysaccharides of *P. cyrtonema* was the highest, at 15.57%.

The results of total polysaccharide, total protein and total polyphenol content in raw polysaccharides of *P. cyrtonema* are shown in Table 1. Among the three raw polysaccharides, the one without fermentation had the highest polysaccharide content, 61.18%, and *P. cyrtonema* fermented with *S. cerevisiae* had the lowest raw polysaccharide content of only 50.04%. All three raw polysaccharides contained polyphenols—the content was similar, below 1%—and they contained a small amount of protein; the content was below 0.5%.

#### 2.1.2. Purification of *P. cyrtonema* Polysaccharide

**Figure 1:**
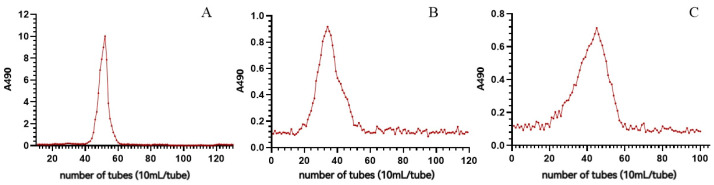

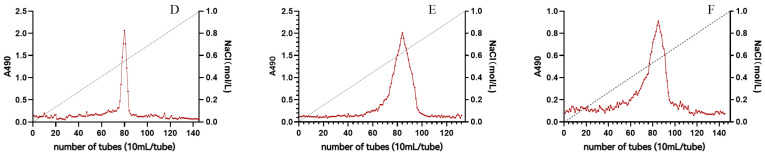
Elution profiles of polysaccharide fractions. (**A**–**C**): RNP, BNP, SNP; (**D**–**F**): RAP, BAP, SAP.

To purify the polysaccharides, three raw polysaccharides were separated using a DEAE-agarose column, and one neutral polysaccharide and one acidic polysaccharide were each obtained. The rates are shown in Table 2, the contents of each component are shown in Table 3, and the elution curves of neutral and acidic polysaccharides are shown in Figure 1. As can be seen from the table, the raw polysaccharide of *P. cyrtonema* was dominated by neutral polysaccharides, accounting for 68.48%; the raw polysaccharide of *P. cyrtonema* fermented by *Bacillus subtilis* was also dominated by neutral polysaccharides, accounting for 64.10%. The difference between neutral and acidic polysaccharides in *P. cyrtonema* fermented by *S. cerevisiae* was smaller, with 47.80% neutral polysaccharides and 52.20% acidic polysaccharides. The polyphenol and protein content of all six pure polysaccharides was less than 1%.

#### 2.1.3. FT-IR Analysis of *P. cyrtonema* Neutral Polysaccharide

**Figure 2:**
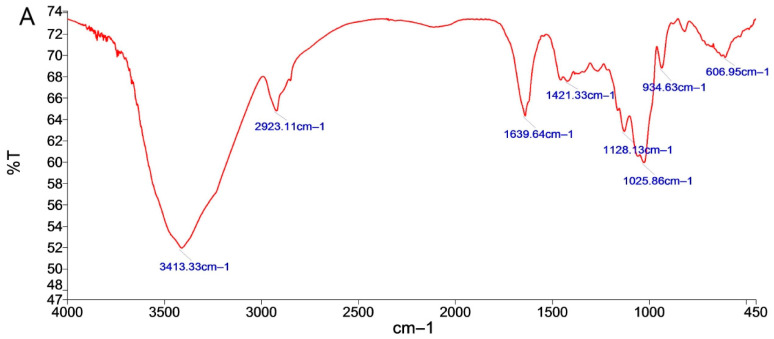

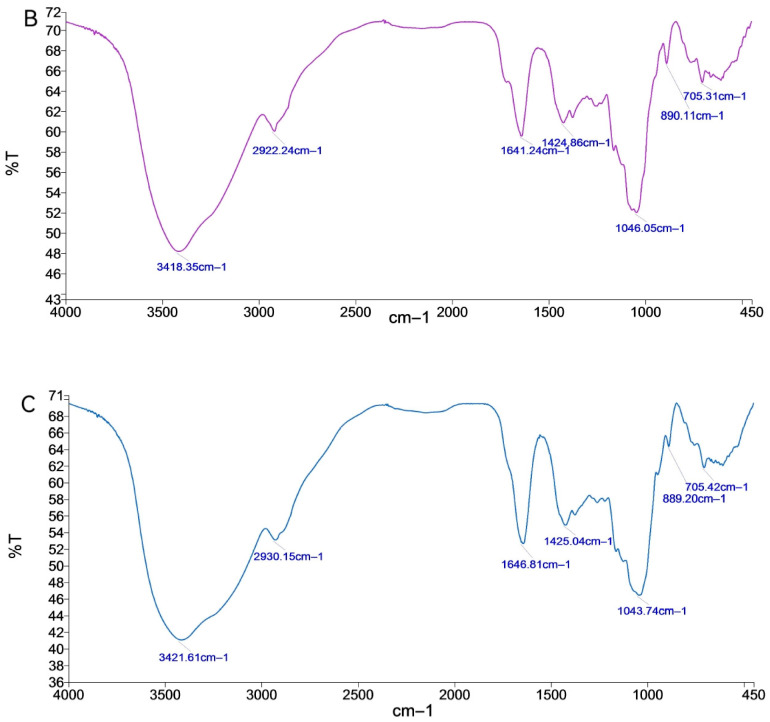
FT-IR spectra of RNP, BNP, SNP: (**A**) RNP, (**B**) BNP and (**C**) SNP.

To preliminarily characterize these polysaccharides, FT-IR was performed. The results of the IR spectra of purified polysaccharides are shown in Figure 2. From the spectra, it can be seen that RNP, BNP and SNP had broad and strong absorption peaks at 3413.33, 3418.35 and 3421.61 cm^−1^ for O-H stretching vibrations in hydrogen bonding [17]. The bands at 2923.11, 2922.24 and 2930.15 cm^−1^ prove the presence of C-H in polysaccharides, and C-H stretching and bending vibrations of CH_2_ and CH_3_ [18]. The absorption peaks at 1639.64, 1641.24 and 1646.81 cm^−1^ are characteristic of protonated carbonyl (C=O) stretching vibrations [19]. The stretching peaks at 1025.86, 1046.05 and 1043.74 cm^−1^ are due to the presence of the C-O bond, indicating the presence of a pyran ring in polysaccharides [20]; monosaccharides are in the form of pyranosides.

The differences in the purified polysaccharides functional groups of the three neutral polysaccharides were shown through the stretching vibration peak of C-O-Cat 1128.13 cm^−1^ for RNP and the characteristic absorption peak of glucose at 934.63 cm^−1^ [21], which were absent for BNP and SNP. The absorption peaks of BNP and SNP at 890.11 and 889.20 cm^−1^ were characteristic absorption peaks of β-glycosidic bonds, which were absent for RNP [22]. RNP did not have this absorption peak. The differences in the infrared spectrograms were able to distinguish the neutral sugars of *C. flavus* before and after fermentation.

#### 2.1.4. FT-IR Analysis of *P. cyrtonema* Neutral Polysaccharides

**Figure 3:**
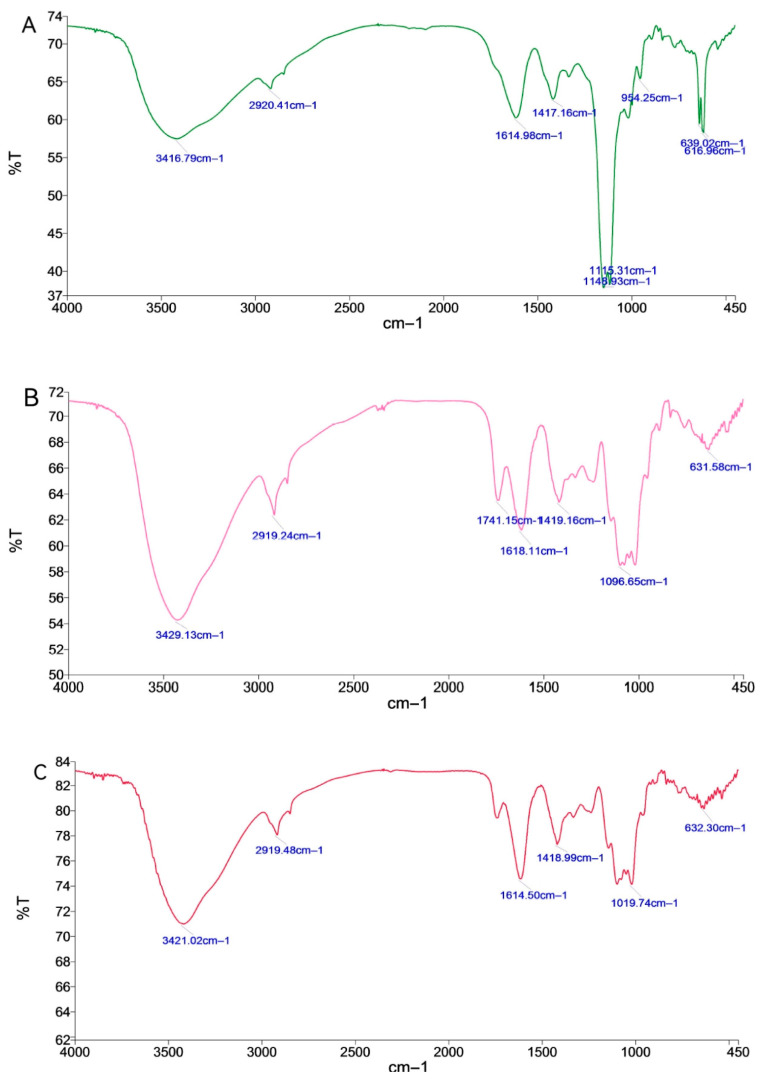
FT-IR spectra of RAP, BAP, SAP: (**A**) RAP, (**B**) BAP and (**C**) SAP.

By analyzing the infrared spectra of acidic polysaccharides (Figure 3), it can be seen that RAP, BAP and SAP all had broad and strong absorption peaks at 3416.79, 3429.13 and 3421.02 cm^−1^ for the stretching vibration of O-H in hydrogen bonding [17]. The bands at 2920.41, 2919.24 and 2919.48 cm^−1^ proved the presence of C-H in polysaccharides, and C-H stretching and bending vibrations of CH_2_ and CH_3_ [18]. Absorption peaks at 1614.98, 1618.11 and 1614.50 cm^−1^ are characteristic of protonated carbonyl (C=O) stretching vibrations [19]. Absorption peaks at 1417.16, 1419.16 and 1418.99 cm^−1^ are the absorption peaks of protonated carboxylic acid COO^-^ groups, and are therefore polysaccharides containing carboxylic acids [23].

The differences between the three acidic glycosyl functional groups of *Flavobacterium aestivum* can be shown by the C-O-C stretching vibration peaks of RAP at 1115.31 cm^−1^ and 1148.93 cm^−1^ [24], generated by the absorption of pyranose cyclic lactones and hydroxyl groups, and the characteristic absorption peak of glucose at 934.63 cm^−1^ [21], which is absent for BNP and SNP. The peak shape of RAP at 1200–600 cm^−1^ is very different from that of BAP and SAP. BAP has a characteristic absorption peak of C=O glucuronide at 1741.15 cm^−1^ [25], which is not present in RAP and SAP. The differences in the peak shapes of the IR spectra could be used to distinguish the acidic polysaccharides of *C. flavus* before and after fermentation easily.

### 2.2. Fermentation Affects the In Vitro Bioactivities of P. cyrtonema Polysaccharides

**Figure 4:**
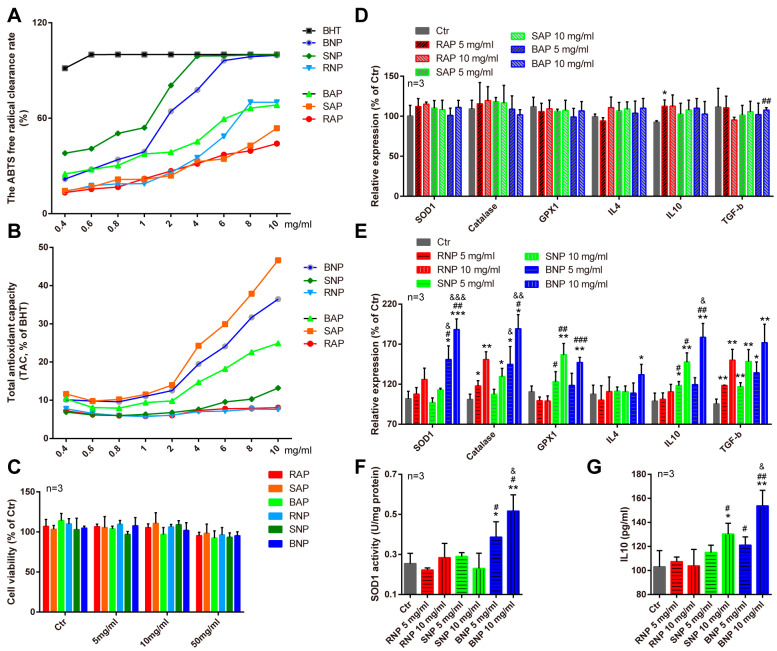
Fermentation affects the in vitro bioactivities of *P. cyrtonema* polysaccharides. (**A**) Quantification showing the rate of ABTS free radical clearance of different *P. cyrtonema* polysaccharides. Butylhydroxytoluene (BHT) was selected as positive control. *n* = 3. (**B**) Quantification showing the total antioxidant capacity of different *P. cyrtonema* polysaccharides. *n* = 3. (**C**) Quantification showing the viability of IPEC-J2 cells treated with different *P. cyrtonema* polysaccharides. (**D**,**E**) Quantifications showing the expressions of common antioxidant genes and inflammatory cytokines in IPEC-J2 cells treated with different *P. cyrtonema* polysaccharides. (**F**) Quantification showing the SOD1 activity of IPEC-J2 cells treated with different *P. cyrtonema* polysaccharides. (**G**) Quantification showing the IL10 levels of IPEC-J2 cells treated with different *P. cyrtonema* polysaccharides. Error bars indicate SD or SEM, * stands for the statistic difference compared with Ctr group, ^#^ stands for the statistic difference compared with the same dose from RNP group and ^&^ stands for the statistic difference compared with the same dose from SNP group. * or ^#^ or ^&^ *p* < 0.05, ** or ^##^ or ^&&^ *p* < 0.01, *** or ^###^ or ^&&&^ *p* < 0.001.

As previous studies have shown, polysaccharides isolated from *P. cyrtonema* with different methods displayed robust antioxidant and immunomodulation capacity [4,26,27]. We first analyzed the effects of fermentation on these bioactivities of *P. cyrtonema* polysaccharides in vitro. With an ABTS assay, which is one of the most widely used assays to evaluate antioxidant property [28], we found that fermentation with both *B. subtilis* and *S. cerevisiae* increased the antioxidant capacity of polysaccharides from *P. cyrtonema*, especially the neutral polysaccharides (BNP and SNP) (Figure 4A). Consistently, an enhanced total reducing capacity of these polysaccharides was also observed using the Total Antioxidant Capacity (TAC) Assay (Figure 4B), the results of which was based on the reduction of ferric iron (Fe^3+^) to ferrous iron (Fe^2+^) [29]. As biomacromolecules, polysaccharides usually cannot be absorbed directly by animals and exhibit their biofunctions through targeting on the intestine. Therefore, we then evaluated the effects of these *P. cyrtonema* polysaccharides on modulating intestinal antioxidant and immune response in cultured intestinal cells, before we tested these in vivo. As shown in Figure 4C, we found no cellular toxicity of polysaccharides from *P. cyrtonema*, with fermentation or without. In addition, we found that the supplementation of neutral polysaccharides from fermented *P. cyrtonema*, especially BNP, could significantly promote the gene expressions of antioxidant enzymes and anti-inflammatory cytokines in cultured IPEC-J2 cells (Figure 4D,E), and these findings were confirmed using ELISA or biochemical assays (Figure 4F,G). All these results reveal that fermentation affects the bioactivities of *P. cyrtonema* polysaccharides, and BNP obtained from *P. cyrtonema* fermented by *Bacillus subtilis* displays attractive bioactivities.

### 2.3. BNP Attenuates Intestinal Oxidative Stress in the Aging Mouse Model More Effectively

**Figure 5:**
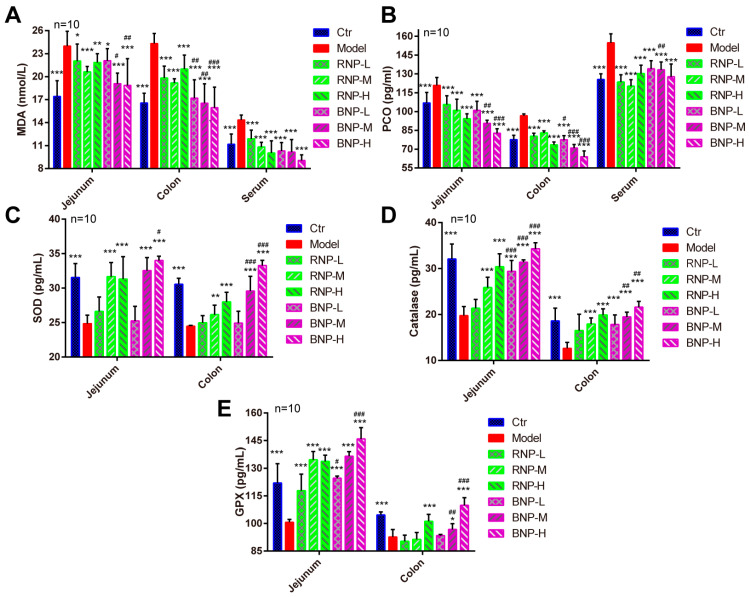
BNP displays better effects on attenuating intestinal oxidative stress in an aging mouse model. (**A**) Quantification showing the malonaldehyde (MDA) levels in jejunum, colon and serum. (**B**) Quantification showing the protein carbonyls (PCO) levels in jejunum, colon and serum. (**C**) Quantification showing the SOD levels in jejunum and colon. (**D**) Quantification showing the catalase levels in jejunum and colon. (**E**) Quantification showing the GPX levels in jejunum and colon. Error bars indicate SD, * stands for the statistic difference compared with Ctr group, ^#^ stands for the statistic difference compared with the same dose from RNP group. * or ^#^ *p* < 0.05, ** or ^##^ *p* < 0.01, *** or ^###^ *p* < 0.001.

Given the differences in the bioactivities among polysaccharides from fermented or original *P. cyrtonema*, we chose BNP, which exhibited most effectively in vitro and RNP from unfermented *P. cyrtonema*, as the control to evaluate the effects of fermentation on the biofunctions of *P. cyrtonema* polysaccharides in vivo. First, we evaluated the antioxidant capacity of these polysaccharides with a D-Galactose-induced accelerated aging mouse model [30], as systemic oxidative stress is a typical symptom of aging [31,32]. By quantifying the levels of malondialdehyde (MDA) and protein carbonyls (PCO), respectively [33], we noticed significantly increased levels of oxidation of lipids and proteins in serum, as well as in the intestine (jejunum from the small intestine and colon from the big intestine), which is thought to be the major target organ of polysaccharides (Figure 5A,B). While we noticed a dose-dependent decrease in the levels of these two products under the supplementation of RNP and BNP, BNP showed a remarkably better capacity compared to RNP, especially in jejunum and the colon (Figure 5A,B). Decreased intestinal oxidative damages could result from increased antioxidative defense induced by RNP and BNP, as we found enhanced levels of important antioxidant enzymes (SOD1, catalase and GPx) in the jejunum and colon from RNP- and BNP-supplied mice (Figure 5C–E), the results of which were consistent with those from cultured IPEC-J2 cells (Figure 4D–G). Also, BNP treatments exhibited more effectiveness in this area. Taken together, these data demonstrated that the fermentation of *P. cyrtonema* with *Bacillus subtilis* could enhance the capacity of neutral polysaccharides from it to promote intestinal antioxidative defense in vivo.

### 2.4. Immunomodulation Effects of BNP in the Immunosuppressed Mouse Model

**Figure 6:**
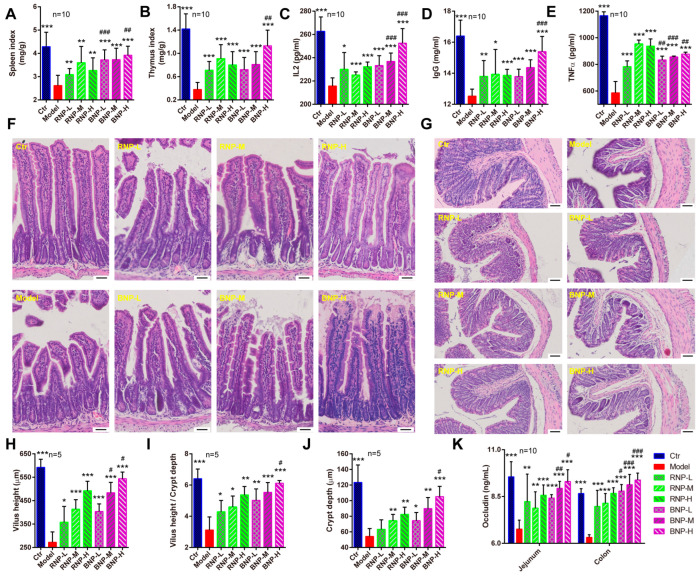
BNP displays better effects on attenuating intestinal defects of immunosuppressed mouse model. (**A**,**B**) Quantifications showing the organ index of spleen and thymus. (**C**–**E**) Quantifications showing the levels of serum of IL2, IgG and TNFα. (**F**) Representative H&E staining images showing the histological structure of the jejunum from different groups. Bar, 50 μm. (**G**) Representative H&E staining images showing the histological structure of the colon from different groups. Bar, 50 μm. (**H**) Quantifications showing the villus height of jejunum from different groups. (**I**) Quantifications showing the ratio of villus height and crypt depth of jejunum from different groups. (**J**) Quantifications showing the crypt depth of colon from different groups. (**K**) Quantification showing the levels of occlusion in jejunum and colon from different groups. Error bars indicate SD, * stands for the statistic difference compared with Ctr group, ^#^ stands for the statistic difference compared with the same dose from RNP group. * or ^#^ *p* < 0.05, ** or ^##^ *p* < 0.01, *** or ^###^ *p* < 0.001.

Immunomodulation is one of the most common effects of polysaccharides isolated from plant herbs, including Polygonati Rhizoma. To evaluate the effects of fermentation on the immunomodulation capacity of *P. cyrtonema* polysaccharides, an immunosuppressed mouse model was generated with cyclophosphamide (CTX) injection [34]. We found that CTX treatment significant decreased the organ index of their two important immune organs, the spleen and thymus (Figure 6A,B). While both RNP and BNP supplementation could rescue the decline of the immune organ index, BNP exhibited more effectiveness, especially in improving the spleen index (Figure 6A). Furthermore, we quantified the serum levels of some typical immune cytokines, which highly correlated with body immunity. We found that RNP and BNP could enhance the levels of serum TNF-α, IL-2 and IgG, suppressed by CTX, in a dose-dependent manner. In addition, middle and high doses of RNP treatment were more effective in improving the serum TNF-α level, while a high dose of BNP treatment was better for improving those of IL-2 and IgG (Figure 6C–E).

**Figure 7:**
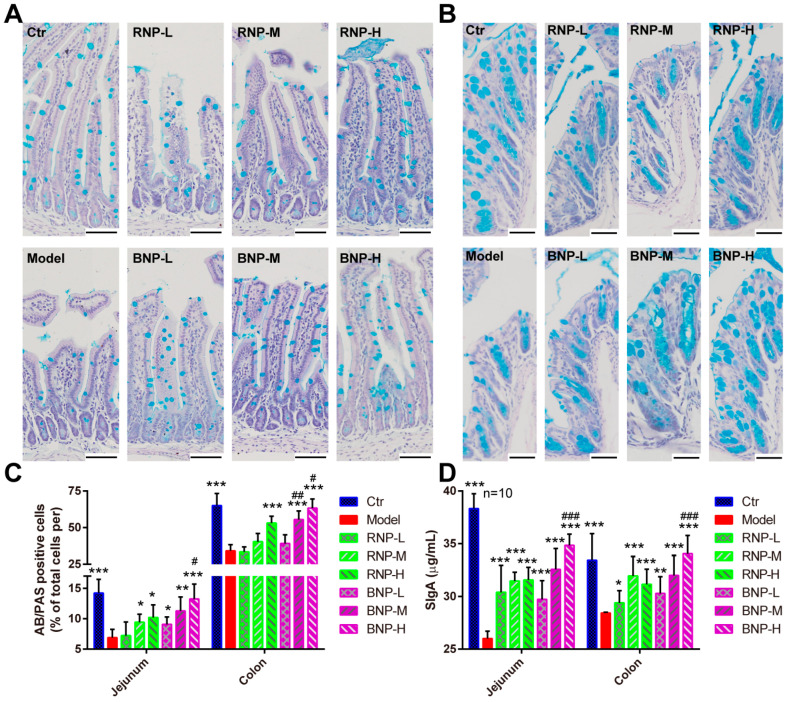
BNP displays better effects on attenuating intestinal immunity of the immunosuppressed mouse model. (**A**,**B**) Representative AB/PAS staining images showing the distribution of goblet cells in jejunum (**A**) and colon (**B**) from different groups. Bar, 50 μm. (**C**) Quantifications showing the relative number of goblet cells in jejunum and colon from different groups. (**D**) Quantification showing the levels of SIgA in jejunum and colon from different groups. Error bars indicate SD, * stands for the statistic difference compared with Ctr group, ^#^ stands for the statistic difference compared with the same dose from RNP group. * or ^#^ *p* < 0.05, ** or ^##^ *p* < 0.01, *** or ^###^ *p* < 0.001.

As the major target organ for polysaccharides is supposed to be the intestine, which is also an important component of the body’s immune system, we then evaluated the effects of RNP and BNP on intestinal histopathological characteristics and immunity. With H&E staining, we noticed the control mice with a regular and orderly arrangement of jejunal villi which were remarkable affected by CTX injection had reduced villi height and reduced ratio of villi height and crypt depth (Figure 6F,H,I), and similar defects of the colon crypt were observed (Figure 6G,J). In addition, robust decreased levels of the tight junction protein occludin were detected after CTX treatment (Figure 6K). After the supplement of RNP or BNP, these defects were improved in a dose-dependent manner, especially in the mice from the high-dose BNP groups (Figure 6H–K). In addition, we used AB-PAS staining to analyze the goblet cells that were important for intestinal immunity, observing significantly fewer goblet cells in the jejunum and colon of CTX-injected mice, while re-balanced numbers of these were found in the mice from RNP- or BNP-supplied groups; BNP showed better effects on this, not surprisingly (Figure 7A–C). Consistent with these benefits, we found that BNP could significantly restore the SIgA level and the expression of intestinal tight junction protein occluding, which were suppressed by CTX injection, better than RNP did in the jejunum and colon (Figure 7D). Therefore, the above results showed the increased capacity of BNP obtained from fermented *P. cyrtonema* in improving the intestinal histological structure and immune function.

### 2.5. BNP Modulates the Homeostasis of Gut Microbiota Affected by CTX in Mice

**Figure 8:**
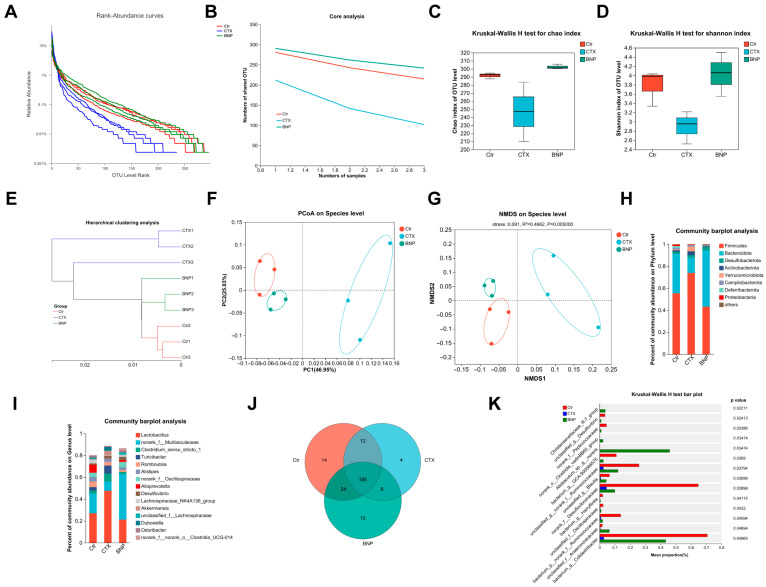
BNP modulates the composition and structure of the gut microbiota of immunosuppressed mice. (**A**) OTU Rank–Abundance curves of gut microbiota for different groups. (**B**) Core OTU analysis among different groups. (**C**) Bacterial community richness measured by Chao1 index among different groups. CTX vs. Ctr, *p* < 0.05, BNP vs. CTX, *p* < 0.05. (**D**) Bacterial community diversity measured with Shannon index among different groups. CTX vs. Ctr, *p* < 0.05, BNP vs. CTX, *p* < 0.05. (**E**) Hierarchical cluster tree showing samples from Ctr, CTX and BNP tend to cluster together. (**F**) Unweighted UniFrac principal coordinate analysis via bacterial microbiota. (**G**) Non-metric multidimensional scaling analysis with bacterial microbiota. (**H**,**I**) Microbial composition at the phylum and genus level. (**J**) Venn diagram showing the unique and shared bacterial species from different groups. (**K**) Quantification showing bacterial species with significantly changed relative abundance among different groups. Only the top 50 bacterial species with the sum relative abundance were calculated.

Increasing evidence is revealing that the gut microbiota is involved in regulating intestinal function and immune response [35,36], and polysaccharides from plant herbs are usually implicated in this response [37,38]. Therefore, we further assessed whether the gut microbiota was implicated in the intestinal benefits of BNP. The gut microbiota composition was analyzed with 16S rRNA V3 + V4 region sequencing, and the rarefaction curves of all samples increased precipitously and then approached a flat level (data not shown), indicating sufficient sequencing depth. Then, with the analysis of Rank Abundance Curve [39], Chao1 index [40] and Shannon index [41], we evaluated the richness and diversity of the intestinal microbiota, observing reductions of them in CTX-treated mice and improvements of them in BNP-supplied mice (Figure 8A,C,D). In addition, the shared OTUs were also reduced in CTX-treated mice and improved in BNP-supplied mice (Figure 8B). To analyze the dissimilarity of the gut microbiota structure among different groups, Abund Jaccard cluster analysis [42] was further performed. We found that samples from Ctr, CTX and BNP group trended to cluster together with those from the same group, and the BNP group clustered closer to the Ctr group (Figure 8E). Consistently, both UniFrac distance-based principal coordinate analysis (PCoA) [43] and non-metric multidimensional scaling analysis (NMDS) [44] showed the distinct clustering of the microbiota composition of mice from the Ctr group compared to those from CTX and BNP groups, while samples from the BNP group clustered much closer to those from the Ctr group than samples from the CTX group (Figure 8F,G). These data revealed a more similar composition of gut microbiota in BNP and Ctr groups.

Then, we focused on the taxonomic distribution of the abundant bacteria from phylum and genus levels in different groups. At the phylum level, *Firmicutes*, *Bacteroidota* and *Desulfobacterota* were most abundant in Ctr (55.4%, 35.8% and 1.9%) and BNP (43.2%, 51.1% and 3.3%) groups, while *Firmicutes*, *Bacteroidota* and *Verrucomicrobiota* were represented most in the CTX group (73.9%, 13.9% and 4.3%) (Figure 8H). At the genus level, *Lactobacillus* (27.0%) was the most enriched genera in the Ctr group, followed by *norank_f__Muribaculaceae* (18.0%) and *alloprevotella* (7.2%). In the CTX group, the abundance of *Lactobacillus* increased to 47.6%, the abundance of *norank_f__Muribaculaceae* decreased to 8.4%, and *Clostridium_sensu_stricto_1* took the third place in the abundance, at 7.4%. On the other hand, in the BNP group, a rebalanced abundance of *Lactobacillus* (21.1%) and *norank_f__Muribaculaceae* (41.7%) were observed, while *norank_f_Oscillospiraceae* (4.8%) was the third most abundant (Figure 8I). To identify the key phylotypes of gut microbiota implicated in the modulation of the intestinal function of BNP, we further compared and calculated the significant difference in the relative abundance of gut microbiota among different groups at species level. As shown in the Venn diagram, we found that 14 species exclusively existed in the Ctr group, and 10 and 4 species were in the BNP and CTX groups, respectively (Figure 8J). Then, the Kruskal–Wallis H test was performed to quantify the differences in the relative abundances of the main bacterial communities at a species level. Among the 50 bacterial species with the highest abundance, 14 showed significant changes in the relative abundance among these three groups. In detail, 11 of these 14 species showed significantly suppressed abundances in the CTX group, while the BNP supplement rescued the decline in an abundance of them. The abundances of the other three species, including *Christensenellaceae*_R-7_group, *norank_o__Clostridia_vadinBB60_*group and *Allobaculum_sp._g__norank*, were particularly increased in the BNP group (Figure 8K). These findings provide evidence showing the effects of BNP on balancing the homeostasis of gut microbiota affected by CTX treatment, which is responsible for its benefits for intestinal function.

## 3. Discussion

Fermentation is becoming one of the most important methods for processing traditional Chinese medicines (TCM) [11]. The fermentation process can improve the solubility and bioavailability of TCM, reduce the toxicity of TCM, and improve the safety and efficacy of TCM. During this process, different kinds of bacteria and fungi play an important role. First, the bacteria/fungi can produce enzymes that promote the metabolism of TCM to reduce their toxicity [11,12]; Second, bacteria/fungi could break down the polysaccharides in TCM, producing substances like acid and alcohol to change the pH value of TCM and releasing some active components, which can improve the efficacy of TCM. Third, the fermentation process with bacteria and fungi can also generate new compounds, which can also affect the pharmacological activities and therapeutic effects of TCM [45,46,47]. For example, the fermentation of Goji juice affects the conversion of the free and bound forms of phenolic acids and flavonoids and improves its antioxidant capacity [48]. Fermented by *Paecilomyces cicadae*, the chemical substances are changed in *Radix astragali*, resulting in an enhanced capacity in ameliorating diabetic nephropathy symptoms [49]. Yan et al. reported an increased anti-diabetic capacity of a fermented Chinese Ge-Gen-Qin-Lian decoction, and Mei et al. found that fermented Shuan-Tong-Ling was beneficial for cerebral ischemia/reperfusion injury through suppressing neuronal inflammation and apoptosis [50]. Apart from these, previous studies have also provided interesting findings on the impact of fermentation on polysaccharide components of TCM [51]. The fermentation of *Tremella aurantialba* produces polysaccharides with the same monosaccharide composition but higher molecular composition [52], and fermentation also enhances the production and antioxidant bioactivities of oolong tea polysaccharides [53]. Lots of bacterium and yeast strains are used in fermentation processes of TCM, and *Bacillus subtilis* and *Saccharomyces cerevisiae* are two of the most commonly used ones. The fermentation of Sanqi with *Bacillus subtilis* produces a new active substance (ginsenoside RH4), and the fermentation of Huangqi with *Bacillus subtilis* results in a much higher polysaccharide content and an increased immune-enhancing effect [54]. *Saccharomyces cerevisiae* contains a variety of vitamins, amino acids and minerals and is notable for its fermentation and functional food applications. Chen et al. reported that *Saccharomyces cerevisiae* fermentation could change the structural characteristics and enhance the antioxidant activity of polysaccharides from wheat bran [55]. In this study, we also found that fermentation has a significant impact on the yield, appearance, content and functional groups of *P. cyrtonema* polysaccharides. Fermentation reduced the yield of crude *P. cyrtonema* polysaccharides, which may be a consequence of their utilization by *Bacillus subtilis* and *Saccharomyces cerevisiae*. For the differences in functional structure, while both neutral and acidic polysaccharides have characteristic functional groups of O-H, C-H, C=O and C-O, with fermentation or without, the absorption peaks of neutral polysaccharides at 1128.13 m^−1^, 934.63 cm^−1^, 889.90 cm^−1^ and 889.20 cm^−1^, and acidic polysaccharides at 954.25 cm^−1^, are differential absorption peaks that undergo transformations due to fermentation. These results suggest that the structure of polysaccharides change due to fermentation, but the detailed structure differences need to be investigated in the future. In addition to their polysaccharide components, our results also displayed impressive effects of fermentation on the bioactivities and biofunctions of *P. cyrtonema* polysaccharides. We noticed that fermented *P. cyrtonema* polysaccharides, especially BNP from *Bacillus subtilis* fermentation, had increased antioxidant capacity themselves and enhanced modulation effects on the intestinal antioxidant defense/immune response in cultured cells. While in vivo, BNP displayed better benefits in attenuating intestinal oxidative stress and modulating its immune response than RNP, which was from unfermented *P. cyrtonema*. All these results indicated that fermentation could be an effective way of processing *P. cyrtonema*, especially for obtaining functional polysaccharides.

Large amounts of polysaccharides are present in the mammalian gastrointestinal tract, while few are directly used as a carbon source and most of the rest are degraded or used by the gut microbiota [56,57]. For this reason, many previous works have demonstrated that the intestine is the target organ of TCM polysaccharides, and modulating the homeostasis of gut microbiota is an important way for TCM polysaccharides to improve lots of diseases [58,59,60,61,62]. In addition, a few reports have revealed interesting effects of fermentation products on gut microtia homeostasis. For example, *Z. rouxii* fermentation products of healthy chicken powder can increase diversity in the foregut microbial community, and fermented Shenling Baizhu San (a traditional Chinese medicine formula) could improve the abundance of *Coprococcus*, *Bifidobacterium* and *Bilophila* in the gut of broilers [63,64]. Here, in this study, we also noticed the function of BNP in modulating the richness, diversity and structure of gut microbiota in an immunosuppressed mouse model, and identified the relative abundance of some bacterial species, such as *Colidextribacter, Ruminococcaceae, Oscillospiraceae, Anaerovoracaceae*, *Blautia*, *Desulfovibrio*, *Desulfovibrionaceae*, etc., which were suppressed through CTX treatment and rescued with BNP supplementation. *Colidextribacter* is thought to be a healthier gut microbiota that functions as an anti-inflammatory probiotic [65,66], and Mager et al. reported benefits of *Colidextribacter* on reducing LPS-induced liver damage and inflammation [67], for example. Similarly, *Colidextribacter*, *Ruminococcaceae* and *Oscillospiraceae* are able to produce secondary bile acids and also function to attenuate inflammation [68,69]. In addition, *Ruminococcaceae* and *Oscillospiraceae*, as short chain fatty acid (SCFA) producers, are also responsible for the degradation of diverse polysaccharides and are closely correlated with diseases linked to intestinal permeability, such as alcoholic cirrhotics, inflammatory bowel disease and non-alcoholic fatty liver disease [70,71,72,73,74,75,76]. Furthermore, *Anaerovoracaceae* is involved in the fermentation of plant polysaccharides in the gastrointestinal tract and is important for intestinal health [77,78], and *Blautia* has been reported to have antibacterial activity and be able to improve inflammatory and metabolic diseases [79,80]. Thus, rebalanced abundances of them in BNP-supplied mice represent the benefits of BNP on intestinal health. Different from the above bacteria, *Desulfovibrio* and *Desulfovibrionaceae*, two sulfate-reducing bacteria, are usually thought to be “bad bacteria” that produce metabolite H_2_S [81,82], which could induce inflammation [83,84]. However, studies have demonstrated that H_2_S can directly promote angiogenesis, which is beneficial for gastrointestinal injures [85], which is consistent with the fact that BNP ameliorates the intestinal histological structure of immunosuppressed mice. In addition to these findings, we also noticed that some bacterial species are particularly enriched in BNP-supplied mice, such as *Christensenellaceae*_R-7_group and *Clostridiales_vadinBB60_* group. The *Christensenellaceae* is reported to be a beneficial gut microbiota and an important player in human health. The relative abundance of *Christensenellaceae* is inversely linked to the host’s body mass index (BMI), metabolic diseases, inflammatory bowel disease, Parkinson’s disease, etc. [86], and these benefits are also reported in the *ChristensenellaceaeR-7* group [87,88,89]. *Clostridiales_vadinBB60_* group is poorly classified, and Hao et al. suggested that this bacterium may play an important role in healthy gut flora [90]. Taken together, the gut microbiota composition analysis reveals that BNP is effective in balancing the homeostasis of gut microbiota, and especially in enriching the abundance of beneficial gut bacteria.

## 4. Materials and Methods

### 4.1. Solid Fermentation of P. cyrtonema

*Bacillus subtilis* (BS01, No. M2012485, preserved in China Center for Type Culture Collection, Wuhan, China) and *Saccharomyces cerevisiae* (purchased from Shanghai Luwei Technology Co., Ltd. (Shanghai, China), strain number ATCC976) were, respectively, activated using solid and liquid Nutrient Broth media and Malt Extract media at a temperature of 37 °C, with a shaking frequency of 120 r/min for *Bacillus subtilis* and 180 r/min for *Saccharomyces cerevisiae*. The activated *B. subtilis* and *S. cerevisiae* were, respectively, inoculated into a liquid culture medium at a ratio of 1:100 and 3:100, and then cultured at 37 °C, 120 r/min (for *B. subtilis*) or 180 r/min (for *S. cerevisiae*) until the late logarithmic growth phase to prepare the fermentation broth under aerobic conditions. *Polygonatum cyrtonema* Hua were purchased for the *Polygonatum cyrtonema* Planting Base of Longchanggou (Ya’an, China), and identified by Pro. Li-Xia Li. The rhizome of *P. cyrtonema* Hua was washed, sliced, steamed and dried at 60 °C. The conditions for the solid fermentation of *P. cyrtonema* were obtained according to the single-factor and optimization of extraction conditions using a Box–Behnken factorial design, based on the isolation rate of total polysaccharide (data not shown). Selected conditions are described below: Firstly, the dried *P. cyrtonema* (~100 g) was mixed with distilled water at a ratio of 1:1 and soaked, until the distilled water was absorbed. Secondly, fully moistened *P. cyrtonema* was cut into particles with a size of 0.2 cm^3^ and steamed for 30 min. Thirdly, the steamed *P. cyrtonema* was dried at 60 ° C until reaching proper moisture content (40% for *Bacillus subtilis* and 28% for *Saccharomyces cerevisiae*), followed by UV irradiation for 30 min. Then, the *P. cyrtonema* were placed in a self-sealed plastic bag as a fermentation substrate, and activated *B. subtilis* fermentation broth with proper ratio (22% for *Bacillus subtilis* and 16% for *Saccharomyces cerevisiae*) was added and mixed. Finally, the air was expelled and the plastic bag sealed, and fermentation was performed at 37 ° C for 41 h (for *Bacillus subtilis*) or 58 h (for *Saccharomyces cerevisiae*). The fermented *P. cyrtonema* were obtained after expelling the gas newly produced during the fermentation.

### 4.2. Extraction, Purification and Preliminary Characterization of Polygonati Rhizoma Polysaccharide

#### 4.2.1. Extraction of Crude Polysaccharides

After solid-state fermentation, products B (from *Bacillus subtilis* fermentation), S (from *Saccharomyces cerevisiae* fermentation) and R (raw material without fermentation) were collected and pulverized to a fine powder with a mechanical grinder (S10, TuoHe Technology Co., Ltd., Shanghai, China) and passed through 0.25 mm mesh (g). Then, 50 g of each material was weighed, respectively, and firstly extracted using ethanol until no color, and residues were collected and further extracted with boiling water, thrice, for 2 h each. The water extracts were combined, concentrated and lyophilized in a freeze dryer (FD-10-50, Beijing Boyikang Experiment Instrument Co., Ltd., Beijing, China). Three crude polysaccharide extracts were obtained and named BP, SP and RP.

#### 4.2.2. Purification of Crude Polysaccharides

Four hundred mg of each crude polysaccharide (BP, SP and RP) powder was dialyzed (cutoff of 3500), redissolved in deionized water, filtered through a 0.45 μm filter, and applied to a DEAE-Sepharose (Fast Flow, FF) column (50 mm × 40 cm, Beijing Rui Da Heng Hui Science Technology Development Co., Ltd., Beijing, China), as described by Huang et al. [91]. Briefly, neutral polysaccharide fractions were firstly eluted with deionized water, the elutes were collected, concentrated and lyophilized, and BNP, SNP and RNP were obtained. Then, acidic polysaccharide fractions were obtained after being eluted with a linear NaCl gradient solution (0–1.5 mol/L, 2 mL/min). The elution solution was collected using a computer automatic collector (5 min per tube and 10 mL per tube). Take 200 μL of the elution solution, mix with 200 μL phenol and 1 mL sulfuric acid; cool and transfer 200 μL for determination using UV spectrophotometry (Multiskan SkyHigh, Thermo Fisher, Waltham, MA, USA) with the absorbance value at 490 nm. Then, use absorbance as the vertical axis and the number of tubes as the horizontal axis to create an elution curve, and merge the required parts based on the elution peak. After pooling eluates together based on the elution profile, concentrating and dialyzing (cutoff 3500 Da), acidic polysaccharide fractions were obtained and named BAP, SAP and RAP, respectively.

#### 4.2.3. Chemical Composition and FT-IR Analysis of Polysaccharide Fractions

Total carbohydrate levels in polysaccharide fractions were determined with a phenol-sulfuric acid assay. The contents of phenolic compounds and protein in all fractions were quantitatively determined using the Folin–Ciocalteu assay [92] and the Bradford protein assay [93], respectively.

The functional groups present in purified fractions were analyzed with Fourier transformed infrared spectroscopy (FT-IR, CREVM/QM03-010, Perkin-Elmer Corp., Waltham, MA, USA). Totals of 10 mg of each polysaccharide fraction and 200 mg of potassium bromide (KBr) were ground and dried under the baking lamp, and then pressed into a 1 mm pellet. KBr was used as a blank for infrared scanning in the range of 4000 cm^−1^ to 500 cm^−1^ (Perkin Elmer, Waltham, MA, USA).

### 4.3. Animals and Treatments

#### 4.3.1. Animals

All mouse work was carried out in accordance with the Animal Care and Use Committee guidelines of Sichuan Agricultural University. All mice were housed in standard, individually ventilated cages under a specific pathogen free (SPF) condition, with 20–22 °C temperature, 12 h light/12 h dark cycle, 50–70% humidity, and ad libitum access to standard chow and water. All the mice, C57/BL6 mice, were ordered from the Vital River Laboratory Animal Technology Co. Ltd., Beijing, China, and one week of acclimatization was performed before they were used for the following studies.

#### 4.3.2. Generation of Aging Mouse Model

Eighty C57/BL6 mice with an age of eight weeks were randomly divided into eight groups. The mice from the model group were subcutaneously injected with D-galactose (B21893, Yuanye, Shanghai, China) (400 mg/kg) for 60 days, while an equivalent amount of physiological saline was given to those from the control group. For the RNP and BNP treatment groups, except for 60 days’ D-galactose (400 mg/kg) injection, different doses of RNP and BNP (high dose, 400 mg/kg/d; medium dose, 200 mg/kg/d; low dose, 100 mg/kg/d) were intragastrically supplied from day 30, and thus named RNP-L, RNP-M, RNP-H, BNP-L, BNP-M and BNP-H, respectively. Physiological saline was intragastrically supplied as a control for the other two groups. After the last injection, the mice were fasted but allowed to drink water overnight, then the blood was collected for further biochemistry assays.

#### 4.3.3. Generation of Immunosuppressed Mouse Model

Eighty C57/BL6 mice with an age of eight weeks were randomly divided into eight groups. Different doses of RNP and BNP (high dose, 400 mg/kg/d; medium dose, 200 mg/kg/d; low dose, 100 mg/kg/d) were intragastrically supplied for 20 days, and the corresponding groups were named RNP-L, RNP-M, RNP-H, BNP-L, BNP-M and BNP-H, respectively. Physiological saline was intragastrically supplied as a solvent control for the other two groups. From day 15, mice from RNP, BNP and one other group (named the model group) were intraperitoneally injected with cyclophosphamide (S30563, Yuanye, Shanghai, China) (80 mg/kg/day) to induce suppressed immunity, while the mice from the last group were Intraperitoneally injected with physiological saline as a solvent control and named the control group. At the end of the experiment, the mice from all groups were fasted overnight and the blood was collected through cardiac blood collection for further assays, and the liver, thymus, spleen, jejunum and colon tissues were collected. At the same time, fresh feces collected from the cecum were stored at −80 °C and sent to Majorbio (Shanghai, China) for gut microbiota analysis.

### 4.4. Biochemical Assay and Enzyme-Linked Immunosorbent Assay (ELISA)

For the biochemical assay of polysaccharides themselves in vitro, indicated concentrations of polysaccharides in PBS were used for the assay. For the biochemical assay or ELISA of serum samples, the blood was kept at room temperature for half an hour after the collection, and then centrifuged at 4000 rpm for 10 min at 4 °C to isolate the serum, which was subjected for further assays. For the biochemical assay or ELISA of tissue samples, ~0.1 g tissue was collected and homogenized in PBS (pH 7.4), and then centrifuged at 2500 rpm for 20 min and the supernatant was collected for further assays. All biochemical assays and ELISA were performed according to the manufacturer’s instructions, and the assay kits used in this study are listed below (Table 4).

### 4.5. Gut Microbiota Analysis

The gut microbiota was analyzed with 16S rRNA gene sequencing. Fecal bacterial DNA extraction, 16S rRNA gene PCR amplification, sequencing and analysis were performed by Shanghai Majorbio Technology Co., Ltd (Shanghai, China). The main methods and steps were as follows: genomic DNA was extracted and the V3-V4 region of the 16S rRNA gene was amplified, and the amplified products were detected and quantified with agarose gel electrophoresis. The purified amplicons were pooled in equimolar amounts and paired-end sequenced on the Illumina MiSeq PE300 platform (Illumina, San Diego, CA, USA). The above sequences were combined and partitioned using the QIIME software to determine the classification level (kingdom, phylum, class, order, family, genus, species) of each sequence. An OTU was defined by a sequence with a similarity greater than 97%. Finally, OTUs were clustered and species were classified based on effective data, and then OTUs were analyzed for abundance and diversity indices. OTU-level alpha diversity, including the Chao1 richness estimator and Shannon diversity index, were calculated to evaluate the abundance and diversity among samples. OTU-level ranked abundance curves were generated to compare the richness and evenness among samples. Beta diversity analysis was performed to investigate the structural variation of microbial communities across samples using UniFrac distance-based principal coordinate analysis (PCoA) and non-metric multidimensional scaling analysis (NMDS). The shared and unique species among groups were visualized using a Venn diagram based on the occurrence of OTUs across groups. Taxa abundances at the species level were statistically compared among groups, and only the top 50 bacterial species with the sum relative abundance were selected in the calculation.

### 4.6. Histological Staining

Organ samples were fixed in 4% paraformaldehyde solution for more than 48 h, then embedded in paraffin. Paraffin sections with a thickness of 5 μm were made and mounted on slides for staining with hematoxylin and eosin (H&E, G1120, Solarbio, Beijing, China) and AB-PAS (G1285, Solarbio, Beijing, China), according to the manufactures’ instructions.

### 4.7. Cell Culture

Intestinal porcine epithelial cells (IPEC-J2) were cultured in Dulbecco’s modified Eagle’s medium (DMEM, Gibco, Waltham, MA, USA) with 10% fetal bovine serum (FBS; Gibco), at 37 °C under 5% CO_2_. To test the effects of different polysaccharides on cell viability, different doses of polysaccharides were subjected to IPEC-J2 cells (~5 × 103 cells per well in 96-well plate) for a 48 h treatment, and the cell viability was evaluated with CCK-8 assay (Dojindo, CK04-11, Minato-ku, Tokyo, Japan) according to the manufacturers’ instructions. To evaluate the effects of different polysaccharides on affecting gene expressions, 5 mg/mL and 10 mg/mL of different polysaccharides were added to IPEC-J2 cells (~1 × 105 cells per well in 6-well plate) for 24 h, then the cells were harvested and subjected to Quantitative Realtime PCR analysis.

### 4.8. Quantitative Realtime PCR

An animal Total RNA Isolation Kit (RE-03014, Foregene, Chengdu, China) was used to extract the tissue total RNA, ~1µg of which was subjected to reverse transcription with the RT EasyTM II kit (RT-01032, Foregene, Chengdu, China) at the following conditions: 42 ℃ for 25 min and 85 ℃ for 5 min. Then, the RT-qPCR was performed using a Bio-Rad CFX96 Real-Time Detection System (Bio-Rad, Hercules, CA, USA) according to the manufacturers’ instructions (RR019B, Takara Biomedical Technology (Beijing) Co., Ltd., Beijing, China), with β-Actin as the internal control to analyze the relative gene expressions. Primers used in this study are listed below (Table 5).

### 4.9. Statistical Analysis

Data represent the mean ± standard deviation (SD) or mean ± standard error of the mean (SEM). One-way ANOVA was performed for the statistical significance analysis using GraphPad Prism software (Version 6.0, San Diego, CA, USA). * *p* < 0.05, ** *p* < 0.01, *** *p* < 0.001.

## 5. Conclusions

In summary, this study validates the effects of fermentation on the components and functions of polysaccharides from *P. cyrtonema*. Solid state fermentation showed an impact on the yield, content, composition, functional groups, etc., of *P. cyrtonema* polysaccharides. After fermentation, the yield of crude polysaccharides decreased, which may be related to the utilization of polysaccharides by fermentation strains. Before and after fermentation, there were differential absorption peaks in the infrared spectra of *P. cyrtonema* polysaccharides between 934.63 cm^−1^, 889.90 cm^−1^ and 889.20 cm^−1^, 954.25 cm^−1^, indicating that fermentation caused changes in the functional groups of *P. cyrtonema* polysaccharides. Furthermore, we report a prominent role of a natural polysaccharide (BNP) from *P. cyrtonema* fermented by *Bacillus subtilis* in modulating the intestinal antioxidant defense to attenuate aging-related intestinal oxidative stress, and in promoting the intestinal immune response to improve intestinal defects of immunosuppressed mice. Furthermore, we found that the beneficial effects of BNP to the intestine may result from its effects in modulating the homeostasis of the gut microbiota. Our findings provide evidence for the further development and utilization of Polygonati Rhizoma, especially its potential in the treatment of intestinal diseases. However, further characterization of BNP is needed to identify the structural basis for its biofunction, which is important for its further applications.

## Figures and Tables

**Table 1:** Yield and composition differences in raw polysaccharides.

Samples	Yield (%)	Polysaccharide Content (%)	Polyphenol Content (%)	Protein Content (%)
R	15.57 ± 0.30	61.18 ± 0.18	0.15 ± 0.01	0.24 ± 0.04
B	12.57 ± 0.52	56.41 ± 0.13	0.67 ± 0.05	0.42 ± 0.03
S	13.22 ± 0.48	50.04 ± 0.23	0.68 ± 0.02	0.28 ± 0.06

Sample R: raw material without fermentation before extraction; sample B: raw material from *B. subtilis* fermentation before extraction; sample S: raw material from *S. cerevisiae* fermentation before extraction. *n* = 3.

**Table 2:** Contents of different components in pure polysaccharide.

Samples	Neutral Component	Neutral Sugar (%)	Acidic Component	Acidic Sugar (%)
RP	RNP	68.48 ± 0.09	RAP	31.52 ± 0.10
BP	BNP	64.10 ± 0.13	BAP	35.90 ± 0.21
SP	SNP	47.80 ± 0.17	SAP	52.20 ± 0.11

RP: raw polysaccharide from water extracts and ethanol precipitation without fermentation; BP: raw polysaccharide from water extracts and ethanol precipitation after *B. subtilis* fermentation; SP: raw polysaccharide from water extracts and ethanol precipitation after *S. cerevisiae* fermentation. *n* = 3.

**Table 3:** Content of purified polysaccharide fractions.

Samples	Polysaccharide Content (%)	Polyphenol Content (%)	Protein Content (%)
RNP	73.90 ± 0.14	0.24 ± 0.04	0.39 ± 0.04
RAP	64.38 ± 0.09	0.18 ± 0.02	0.42 ± 0.02
BNP	68.51 ± 0.15	0.32 ± 0.04	0.53 ± 0.05
BAP	59.87 ± 0.12	0.21 ± 0.01	0.47 ± 0.06
SNP	68.69 ± 0.17	0.26 ± 0.02	0.35 ± 0.02
SAP	65.24 ± 0.14	0.15 ± 0.02	0.29 ± 0.02

RNP and RAP: neutral and acidic polysaccharide from un-fermented herbs; BNP and BAP: neutral and acidic polysaccharide from *B. subtilis* fermentation, SNP and SAP: neutral and acidic polysaccharide from *S. cerevisiae* fermentation. *n* = 3.

**Table 4:** Assay kits used in this study.

Name	Company	Article Number
Antioxidant Capacity Assay Kit with ABTS	Mlbio (Shanghai, China)	ml094998
TAC assay kit	Mlbio (Shanghai, China)	ml076332
IL-2 ELISA kit	Mlbio (Shanghai, China)	ml063136
sIgA ELISA kit	Mlbio (Shanghai, China)	ml001917
IFN-γ ELISA kit	Mlbio (Shanghai, China)	ml002277
GSH-Px ELISA kit	Mlbio (Shanghai, China)	ml058194
Occludin ELISA kit	Mlbio (Shanghai, China)	ml063481
SOD activity assay kit	Mlbio (Shanghai, China)	ml095266
SOD ELISA kit	Mlbio (Shanghai, China)	ml643059
IgG ELISA kit	Mlbio (Shanghai, China)	ml037601
TNF-α ELISA kit	Mlbio (Shanghai, China)	ml002095
MDA assay kit	Mlbio (Shanghai, China)	ml094963
Catalase ELISA kit	Mlbio (Shanghai, China)	ml037752
PCO ELISA kit	Mlbio (Shanghai, China)	ml058321

**Table 5:** Primers used in this study.

Gene	Primer	Sequence
*β-actin*	F	5′-CATCCTGCGTCTGGACCTGG-3′
	R	5′-CAATAGTGATGACCTGGCCGT-3′
*IL-4*	F	5′-TTCGGCACATCTACAGACACC-3′
	R	5′-TTCATGCACAGAACAGGTCA-3′
*IL-10*	F	5′-CTGCCTCCCACTTTCTCTTG-3′
	R	5′-TCAAAGGGGCTCCCTAGTTT-3′
*TGF-β*	F	5′-AGGGCTACCATGCCAATTTCT-3′
	R	5′-CCGGGTTGTGCTGGTTGTACA-3′
SOD1	F	5′-ACCTGGGCAATGTGACTG-3′
	R	5′-TCCAGCATTTCCCGTCT-3′
Catalase	F	5′-AACTGTCCCTTCCGTGCTA-3′
	R	5′-CCTGGGTGACATTATCTTCG-3′
GPX1	F	5′-CGGACCACCTGTTGAAAGCTC-3′
	R	5′-TCCGCCAGTTCTTGTTGTCCA-3′

## Data Availability

Source data are provided in this paper and are available from the corresponding author upon reasonable request.
